# A Study of Using Metaphoric and Beat Gestures with Motion-Based and Non-Motion-Based Metaphors during Retelling Stories

**DOI:** 10.3390/bs12050129

**Published:** 2022-04-29

**Authors:** Omid Khatin-Zadeh, Danyal Farsani, Florencia Reali

**Affiliations:** 1 School of Foreign Languages, University of Electronic Science and Technology of China, Chengdu 610054, China; khatinzadeh.omid@yahoo.com; 2 Facultad de Educación, Psicología y Familia, Universidad Finis Terrae, Santiago 7501015, Chile; 3 Department of Psychology, Universidad de los Andes, Bogotá 111711, Colombia; f.reali96@uniandes.edu.co

**Keywords:** metaphoric gestures, motion-based metaphors, static event-based metaphors, static object-based metaphors, static space-based metaphors

## Abstract

In this paper, we classify metaphors into four categories: motion-based metaphors, static space-based metaphors, static object-based metaphors, and static event-based metaphors. Then, a study that investigated the use of gestures with these types of metaphors is reported. The aim was to examine how these types of metaphors are used with metaphoric and beat gestures during the process of re-telling stories. The participants of the study listened to three audio stories. Each story contained two motion-based metaphors, two static space-based metaphors, two static object-based metaphors, and two static event-based metaphors. After listening to each story, they had to retell the stories in front of a camera. The videos were analyzed to determine the number of metaphoric gestures and beat gestures that had been used by participants during the retelling of the stories. The results showed that the highest number of metaphoric gestures had been used with static space-based metaphors. This was followed by motion-based metaphors, static object-based metaphors, and static event-based metaphors, respectively. On the other hand, the highest number of beat gestures was used with static event-based metaphors. These findings indicate that the use of metaphoric gestures and beat gestures accompanying metaphors is highly dependent on the spatial and motoric properties of the base of the metaphors, which supports the idea of embodied metaphor comprehension.

## 1. Introduction

Metaphor and metaphoric gestures are important parts of language and daily communication. Although metaphors are a verbal mode of communication and metaphoric gestures are a non-verbal mode, both share some intrinsic features. Metaphor and metaphoric gestures belong to the figurative aspects of language. Both describe something beyond its literal features. According to Lakoff and Johnson, the essence of a metaphor is understanding something in terms of another thing, usually an abstract less familiar domain in terms of a concrete more familiar domain [1]. The concrete more familiar domain and the abstract less familiar domain are called the base and target of the metaphor, respectively. A metaphoric gesture that is used with a metaphor offers an embodied visual description of the base of the metaphor. In recent years, a growing number of studies have demonstrated that metaphoric gestures can enhance people’s comprehension and learning processes [2,3,4]. This gestural description does not illustrate the literal features of the target of the metaphor; rather, a metaphoric gesture typically illustrates the image of the base of the metaphor. There is some strong evidence from gesture studies suggesting that metaphors and abstract concepts described metaphorically are grounded in embodied experience, e.g., [5,6,7,8,9]. 

In this paper, a model for classifying metaphors is proposed. This is followed by a review of various types of gestures with a focus on metaphoric gestures. Additionally, the ways that metaphoric gestures are used with these metaphors are discussed. Then, an experiment is reported in which the use of metaphors and metaphoric gestures during the retelling of a set of stories was examined. The aim of this experiment was to investigate how metaphors and metaphoric gestures are employed by the speakers during the retelling of stories. In particular, this study explored whether participants’ use of metaphoric gestures was associated with the retelling of certain types of metaphors over others. 

## 2. A Classification of Metaphors

Based on a variety of characteristics, metaphors can be classified into different categories. Here, we propose a classification of metaphors on the basis of features of the base of metaphor. We limit the classification to metaphors that describe an abstract concept in terms of a concrete concept. This classification is based on whether the base of a metaphor is a motion-related or a non-motion-related concept. In the past works that have been conducted on metaphors, motion-related and non-motion-related metaphors have been discussed generally, and no distinction has been made between various types of these metaphors. Making a distinction between various types of these of metaphors could offer a clearer picture of how these metaphors are used and processed. We categorize metaphors into four types:

(1) Motion-based metaphors;
(2) Static space-based metaphors;
(3) Static object-based metaphors;
(4) Static event-based metaphors.

A motion-based metaphor is a metaphor that describes an abstract concept as a motion event [10]. The metaphors ‘time is moving fast and the date of final exams is approaching’ conceptualize the concept of time as a moving object. The metaphor ‘he fell down into a state of depression’ describes the psychological state of depression as a downward movement. Sometimes details of movement such as the speed and direction of movement (fast movement, upward movement, etc.) are used in these metaphors. Static space-based metaphors describe an abstract concept in terms of a location in the space. The metaphors *he is in a* ‘high position in our organization’ and ‘they are living at the lowest level of society’ describe abstract concepts in terms of locations in the space. These metaphorical descriptions do not involve any movement. This is why we call them static space-based metaphors. Static object-based metaphors describe abstract concepts in terms of static objects. The physical characteristics of base object such as size, shape, and other features may be involved in this category of metaphors. The metaphor *strict regulations are a big obstacle for our company* describe strict regulations as an obstacle. The metaphor *my father’s way of life was a candle for me* describes a father’s way of life as a candle that produces light and shows the right direction. Static event-based metaphors describe an abstract concept in terms of a static event. The metaphor *his words revolutionized my way of thinking* describes a change in the way of thinking in terms of a revolution. Here, revolution is a static event. It should be noted that a revolution may involve some degree of motion associations. However, these associations are not central to the metaphorical meaning. The core meaning of this metaphor is the concept of change. Therefore, this metaphor is included in the category of static event-based metaphors in our classification of metaphors. To take another example, in the metaphor *achieving this goal is a piece of cake*, a concept is understood as eating a piece of cake. The main part of the meaning of this metaphor is the easiness of doing something. Although the action of eating a cake involves some degree of movement (e.g., movement of jaws), these movements are not central to the metaphorical meaning. Therefore, this metaphor is included in the category of static event-based metaphors.

## 3. McNeill’s Classification of Gestures

Like metaphors, gestures can be classified into a variety of categories, e.g., [11,12] depending on the characteristics that distinguish between different types of gestures. McNeill’s typology [12] of gestures is one of the most well-known classifications. According to this classification, gestures are categorized into four main types: (1) pointing (deictic) gestures, (2) iconic gestures, (3) metaphoric gestures, and (4) beat gestures. Pointing gestures are used to refer to objects or locations in the surrounding environment. This type of gesture usually takes the form of an extended index finger. However, other fingers or even the entire hand may also be used as a pointing gesture. In this type of gesture, the shape of the finger or hand does not have any semantic relationship with the object or the locations it refers to. Iconic gestures illustrate the shapes of objects by the shapes of hands or the trajectory of hand movements. Iconic gestures have an iconic relationship with the shape or semantic content of objects they refer to. For example, tracing the shape of a circle in the air can be used to refer to a circle. Metaphoric gestures are used to refer to the metaphorical meaning of concepts. For example, a grasping gesture can refer to the understanding of an idea (the metaphor *grasp an idea*). In fact, a metaphoric gesture illustrates the image of the base of the metaphor. In the metaphors *I grasped the idea*, the understanding of an idea (target of the metaphor) is metaphorically described in terms of grasping a concrete object (base of the metaphor). The metaphoric gesture used with this metaphor (a grasping gesture) depicts the base of this metaphor. Beat gestures are gestures that are aligned with the prosody or structure of speech. Beat gestures accompany speech, but they do not express any semantic information. Iconic and metaphoric gestures have been called representational gestures [12]. Representational gestures illustrate either literal or metaphorical aspects of concepts or objects [13,14]. In addition to these types of gestures, interactive gestures are also used in conversational interactions. Interactive gestures help conversational parties manage turn taking during conversation. For example, in a conversation, one of the parties may use a gesture to tell the other party that it is her/his turn to talk about an issue. Like beat gestures, interactive gestures do not express any semantic information.

## 4. Mental Processes Involved in Metaphoric Gestures

A number of models have been proposed by researchers to describe mental processes involved in gesture production, such as the sketch model [15], the lexical gesture process model [16], the interface model [17], the growth point theory [12,18], and the gesture-in-learning-and-development framework [19]. At a general level, all these models share the view that gestures emerge from spatial and motoric properties. Among the models that have been suggested to describe gesture production, perhaps one of the most influential ones is gesture-as-simulated-action, according to which gestures arise from perceptual and motor simulations that take place in the mind [20,21]. In other words, they view gestures as the reflection of embodied simulations. Based on this model, spatial representations and mental images underlie gestures. It has been argued that when a concept or an idea is described or simulated in terms of perceptual or motor properties, a gesture may be produced, regardless of whether that concept is really (literally) spatial or metaphorically spatial [20]. In other words, even when a concept is metaphorically understood as a motion event, the motion is simulated in the mind of the individual, and this simulation may result in a metaphorical gesture. This assumption of the gesture-as-simulated-action framework is similar to the view that is held by the strong versions of embodied cognition [22], according to which the same neural networks that are involved in the processing of the base of a metaphor are also involved in the processing of the target of that metaphor. For example, the same neural networks that are activated during processing the concept of “grasping” are employed to process the metaphor *I grasped the idea*. It has been argued that the same neural networks that are involved in the action of grasping are activated when the individual thinks about the action of grasping and even when s/he comprehends the metaphor *I grasped the idea* [23]. These key claims of the strong versions of embodied cognition have been supported by a range of works, at least to some extent, e.g., [23,24,25,26,27,28]; for a review, see [29].

Regarding the processing and producing of metaphoric gestures, the similarity of views between the gesture-as-simulated-action framework and the strong versions of embodied cognition has some important implications. The gesture-as-simulated-action framework holds that every metaphoric gesture is the result of a simulation of sensorimotor features that initially takes place in the premotor areas. When this simulation or activation in the premotor areas surpasses gesture threshold and spreads to motor areas, a metaphoric gesture is produced [20]. Similarly, the strong versions of embodied cognition claim that when a metaphor that describes an abstract concept in terms of body movements is used by a speaker or comprehended by a listener, the sensorimotor networks that are involved in the production of these body movements are activated. This activation initially takes place in the premotor areas and may spread to motor areas, if the level of activation in the premotor areas is strong enough. This shared view of the gesture-as-simulated-action framework and the strong versions of embodied cognition is supported by the findings of a study that examined the impact of real and imagined body movements on metaphor comprehension. In this study, Wilson and Gibbs [9] found that when people made real body movements related to a metaphorical phrase or even imagined body movements related to a metaphorical phrase, their immediate comprehension of thrse phrases was facilitated. For example, making a pushing gesture or imagining pushing movements facilitated the understanding of the metaphorical phrase *push the argument*. Evidence supporting the idea of embodied metaphor comprehension has been provided by a number of other studies [30,31,32,33,34,35]. Gibbs [36] has advanced the idea of embodied metaphor comprehension and argued that metaphor production and comprehension are a part of a dynamical–ecological process and “must always be characterized as embodied, enactive, embedded, and extended” (p. 33). The idea of metaphor comprehension as a dynamical–ecological process has been discussed in several works, e.g., [37,38,39]. 

Here, we report a study that examined embodied metaphor comprehension on the basis of our proposal for classifying metaphors. In this study, we examined the association of the use of metaphoric gestures with motion-based metaphors, static space-based metaphors, static object-based metaphors, and static event-based metaphors during the retelling of three stories. Following shared views of embodied cognition theories and gesture-as-simulated-action framework, an association between the use of metaphoric gestures and motion-based metaphors was expected.

## 5. Method

### 5.1. Participants

Twenty-seven undergraduate university students from Chabahar Maritime University participated in this study. They were 19–24 years old, and the sample included 16 females and 11 males. All participants were Persian native speakers. 

### 5.2. Materials

Three short audio stories were used in this study, each one was around 5 min and included about 550 words. These stories were in Persian. The first story was about a child labor victim who later became a successful writer. The second story was about an ordinary laborer who managed to become a top manager. The third story was about a woman who experienced a bad event that turned out to have very good consequences. Each story contained 8 metaphors (2 motion-based metaphors, 2 static space-based metaphors, 2 static object-based metaphors, and 2 static event-based metaphors). The order of the type of metaphors was randomized across the three stories. The contents of the stories were carefully checked by the researchers to make sure that only eight metaphors had been included in each story. The English translations of metaphors are shown in the Appendix A. This is the translation of an extract of one of the stories:

“She had enthusiastically prepared herself for the interview. She believed she would finally get the job because she had all needed qualifications. However, results of the interview was like a shock. She failed. After this unexpected failure, she fell down into a state of depression. She believed she deserved to get the job, but she had been treated unfairly”. 

### 5.3. Procedure

Before conducting the experiment, participants attended a training session designed to familiarize them with the procedure of the experiment. In this training session, a sample story was presented to the participants, and they were asked to retell it in front of a camera. However, the aim of the study was not revealed to them. Additionally, participants were provided with oral instructions just before the onset of the experiment. During the experiment, participants sat in front of a computer screen. First, they listened to the first audio story. After listening to it, they immediately turned on the camera of their computers and retold the story in their own language. They had 5 min to retell the story. The distance between each participant and the camera allowed the camera to record the gestures that were produced by participants during retelling. Participants had to provide as much details as they could during the retelling of the story. The same procedure was used for the other two stories. Each participant listened to the three stories. The order of presentation of stories was the same for all participants. 

### 5.4. Data Analysis

The video recordings of participants’ reproductions of the stories were analyzed by the researchers of the study. In this analysis, the researchers of the study examined the metaphoric gestures that accompanied each type of metaphor. For example, a metaphoric gesture could be used with a motion-based metaphor during the retelling of the story. The metaphors produced during retelling the stories were counted. This was carried out for each type of metaphor separately. Additionally, the numbers of metaphoric gestures and beat gestures produced with each type of metaphor were obtained. A metaphoric gesture was defined as the movements of hand(s) accompanied by a verbal metaphor to express a meaning metaphorically. A beat gesture was defined as the movements of hand(s) that were aligned with the prosody and the structure of the accompanying speech. The coding and analyzing of metaphors and gestures was carried out by the leading researcher of the study. This was conducted two times by the same researcher. The value of intra-rater reliability was 1.

## 6. Results

The sums of produced metaphors during the retelling of the stories are presented in Table 1. The sums are shown separately for each type of metaphor. Additionally, the number of metaphoric gestures and beat gestures that accompanied each type of metaphor are shown in this table.

Table 1 shows that metaphoric gestures accompanied static event-based metaphors to a lesser extent than the other three types of metaphor. A contingency table analysis showed a significant association between the frequency of metaphoric gestures and type of metaphor (χ^2^ (3, n = 468) =159, *p*< 0.001), suggesting that participants tended to use significantly fewer metaphoric gestures when they used static event-based metaphors. Conversely, the number of beat gestures that accompanied static event-based metaphors was significantly larger than the number of beat gestures that accompanied the other three types of metaphor (χ^2^ (3, n = 468) =36.5, *p* < 0.001).

## 7. Discussion

The results of this study showed that participants actively used all four types of metaphors during the retelling of the stories. However, the number of metaphoric gestures that accompanied static event-based metaphors was significantly smaller than the numbers of metaphoric gestures that accompanied the other three types of metaphor. Conversely, beat gestures were more frequently used with static event-based metaphors compared to the other three types of metaphor. These results suggest that in terms of using metaphoric gestures, static event-based metaphors have some characteristics that make them different from other types of metaphor. One possible difference between static event-based metaphors and the other three types of metaphor is the way that information is distributed between speech and gesture. The results of this study suggest that when static event-based metaphors are used, the intended message is primarily expressed by speech. Even when gestures are used with these metaphors, they are mostly beat gestures, which carry little or no information at all. Some static event-based metaphors involve details that are difficult to illustrate by gestures. For example, the Persian metaphor *he was drowned in his thought* describes “deep thinking” in terms of “being drowned in water”, which is a non-motion event. This metaphorical description does not involve a noticeable movement, a spatial element, or an object with an easily illustratable shape. Therefore, it is unlikely that using a gesture can help the speaker to express a significant part of semantic content. Instead of using metaphoric gestures, some Persian speakers use a beat gesture with this metaphor. This was clearly observed in the data that were collected in this study. A question that may be raised here is why beat gestures are used mostly with static event-based metaphors. To answer this question, it should be noted that static event-based metaphors such as *his words revolutionized my way of thinking* often involve less salient spatial elements. Therefore, metaphoric gestures cannot be very helpful in expressing information. Instead, beat gestures are used to support the process of recalling non-spatial information related to the event. The role of beat gestures in recalling information is supported by some evidence provided in the past works, e.g., [40]. 

To take another example, the metaphor *he is now in peace* describes “death” in terms of “being in the psychological state of peace”, which is a non-motion event. Similar to the previous example, this metaphor, which is a static event-based metaphor, does not involve a noticeable motion, a significant spatial element, or an object with a clear shape. Therefore, not much information of this metaphor can be expressed by gestures. On the other hand, the metaphor *he left us alone* describes the concept of “death” in terms of “moving away from somebody”. This metaphor is more likely to be used with a gesture, as movements and spatial elements involved in it are easy to illustrate in terms of gesture. This is also the case with static space-based metaphors. Although these metaphors do not involve movements of objects, their bases have easily illustratable shapes. This allows the speaker to easily show the base of the metaphor with a gesture when s/he uses the metaphor. In fact, the speaker simulates the process of formation of the base. In this way, a static base is transformed into a motion-based representation, and the static object-based metaphor is transformed into a motion-based metaphor. Based on the strong versions of embodiment [22], using these metaphors by a speaker and even comprehending them can activate the motor system. From this perspective, when a speaker uses these metaphors, s/he simulates the movements that are involved in them. This mental simulation is caused by the active involvement of the motor system. The activation of the speaker’s motor system can play a causal role in producing gestures when the speaker uses a motion-based metaphor or a static object-based metaphor. The same thing may happen when static space-based metaphors (e.g., *she has a top position in our department*) are used by a speaker or even comprehended by a listener. In these metaphors, the relative location of static objects in the space is simulated in the mind. Similar to the simulation that is conducted during using static object-based metaphors, the simulation of relative locations of objects in the space can involve movement. This simulation that takes place by the active role of the motor system can be a strong push for the speaker to produce a gesture with these metaphors.

The key point about motion-based metaphors, static space-based metaphors, and static object-based metaphors is that in these three types of metaphor, an abstract concept is embodied as a motion or location in the space, which are simulated in the mind and its concrete realization is shown by the gesture. In fact, the initial mental simulation that takes place in the mind could be the primary reason behind using gestures with these types of metaphor. As Hostetter and Alibali argue [20,21], gestures arise from motor simulations that underlie mental imagery. When a speaker uses a motion-based metaphor, a static space-based metaphor, or a static object-based metaphor, a motor simulation takes place that creates a mental image of a motion, a location in the space, or a shape. This motor simulation, which is entirely an internal or mental process, may be realized in the form of gestures. Gestures may then arise from the mental simulation of a concrete or an abstract concept. Regardless of whether the concept that is being described is abstract or concrete, it is embodied as a motion or space-situated image, and it is shown by gestures. Along the same line, it has been proposed that conceptual metaphors map abstract concepts into physical concepts that can be depicted with gestures [41]. 

The use of metaphoric gestures with metaphors can also be dependent on the extent to which a metaphor is schematic. Some metaphors are highly schematic. For example, the metaphor *the lack of budget was an obstacle that hampered the rapid movement of the company toward its goals* is highly schematic. The schema of this metaphor can be shown as an object that prevents the movement of another object. The metaphor *a new challenge rose up in front of us* is another metaphor that is highly schematic. The schema of this metaphor can be shown as the rising of an object on a surface. The schema of each one of these metaphors, which is the key part of the metaphor, can be easily illustrated by gestures. This easiness can be a good motivation for the speaker to use metaphoric gestures with these metaphors, as a major part of metaphorical meaning can be encoded in the metaphoric gestures.

## 8. Limitations of the Study and Future Directions

In the course of conducting this study, we were faced with two main limitations. Due to difficulty of access to the participants, this study was conducted on a small population. If the study had been conducted on a larger number of participants, more reliable results could have been obtained [42]. Additionally, because of the difficulty of accessing people from a variety of cultures, participants of the study were selected from only Persian native speakers. Since culture and linguistic background are two important factors that can affect the use of metaphor and gesture, the scope of our study was limited in this respect [43]. If participants of the study had been selected from a variety of cultures, more comprehensive and reliable results could have been obtained. In future research projects, replicating this study on larger populations from a variety of cultural and linguistic backgrounds can produce more comprehensive and informative results.

## 9. Conclusions

Based on the results of this study, it is concluded that the type and number of gestures that accompany metaphors are, at least partially, dependent on the types of metaphors. Among the four types of metaphors that were investigated, motion-based metaphors, static space-based metaphors, and static object-based metaphors are more likely to be used with metaphoric gestures, while static event-based metaphors are more likely to be used with beat gestures. One possible reason for this difference is the way that information is distributed between speech and gesture. Due to their nature, when a motion-based metaphor, a static space-based metaphor, or a static object-based metaphor is used, the core part of the information can be expressed by metaphoric gestures. Another possible reason for this is based on the ways that concepts are simulated in the mind of the speaker. The mental simulation that takes place during the use of motion-based metaphors, static space-based metaphors, and static object-based metaphors usually involves motion and the activation of the motor system. The activation of the motor system can be the cause of producing gestures when these types of metaphor are used. 

## Figures and Tables

**Table 1:** Total counts of produced metaphors and accompanying gestures for each type of metaphor.

Type of Metaphor	Motion-Based Metaphor	Static Space-Based Metaphor	Static Object-Based Metaphor	Static Event-Based Metaphor
Number of produced metaphors	138	122	101	107
Number of produced metaphoric gestures	103	109	72	15
Number of produced beat gestures	11	7	5	29

## Data Availability

Data sharing not applicable.

## Appendix A

English translations of metaphors used in the stories

**Motion-based metaphors**

1. He rose up in the hierarchy of the organization
2. People’s morale went up
3. Time is moving fast
4. She fell down into a state of depression
5. We are approaching Christmas holidays
6. She left this world at a young age

**Static space-based metaphors**

1. She is in a high position in our company
2. They are living at the lowest level of society
3. They were looking at the situation from two different perspectives
4. His friend thought highly of him
5. A large part of society is living under the line of poverty
6. His book stands among top novels

**Static object-based metaphors**

1. He had a heavy influence on his friends
2. A writer’s pen is stronger than a dictator’s sword
3. He is living in a sea of problems
4. Your advice is a lighthouse for me
5. His father’s way of life was a lighthouse for him
6. Strict regulations are a big obstacle for our company

**Static event-based metaphors**

1. He was simmering in anger
2. The man was drowned in his thoughts
3. She managed to conquer her illness
4. After hearing the bad news, he was destroyed
5. He is now in peace
6. His words revolutionized my way of thinking
